# Evaluating the scotopic visual sensitivity of walleye (*Sander vitreus*) and implications for foraging habitat

**DOI:** 10.7717/peerj.21156

**Published:** 2026-04-29

**Authors:** Trevor D. Keyler, Loranzie S. Rogers, Thomas R. Hrabik, Kelly A. Harrington, Allen F. Mensinger

**Affiliations:** 1 Biology, College of St. Benedict and St. John’s University, Collegeville, MN, United States; 2 Biology, University of Minnesota–Duluth, Duluth, MN, United States; 3 Harvard University, Cambridge, MA, United States; 4 Dotmatics, Boston, MA, United States

**Keywords:** Scotopic vision, Electroretinography, Visual habitat, *Sander vitreus*, Light attenuation, Freshwater fish

## Abstract

Understanding the physiological limits of visual detection is essential for delineating habitat use in freshwater fishes. Walleye (*Sander vitreus*), a crepuscular predator with well-documented low-light foraging capabilities, exhibits visual adaptations suited for dim environments. Using electroretinography (ERG), we determined the scotopic spectral sensitivity of adult *S. vitreus* and modeled their potential visual foraging depth under a range of optical conditions. Peak spectral sensitivity occurred between 500–550 nm, aligning with wavelengths that penetrate mesotrophic systems most effectively. We used ERG-derived irradiance thresholds in combination with the Beer-Lambert light attenuation model to estimate maximum depths for visual detection under solar and lunar illumination across a range of turbidity levels. Results indicate that under daylight conditions, *S. vitreus* can detect light to depths exceeding 77 m in clear water (kPAR = 0.3) and ~13 m in turbid systems (kPAR = 1.2). Under moonlight, detection is possible to 11.3 and 1.9 m, respectively. These depth estimates exceed commonly reported habitat use, suggesting that vision may remain functional beyond expected depth ranges. These results provide a physiological basis for understanding the depth limits and illumination conditions under which scotopic vision may remain functional in walleye across freshwater systems of varying water clarity (kPAR).

## Introduction

Fishes inhabit a broad range of photic habitats in which selective pressures have driven various visual adaptations (Collin, 1997). For example, latitude, season, time of day, and the optical properties of water modulate downwelling light available in aquatic ecosystems, thereby shaping the spectral composition and intensity of light available for visually mediated behaviors (Horodysky et al., 2010). Fishes, therefore, have evolved visual systems that are well adapted to the light environment within their respective habitats and temporal niche (*i.e*., diurnal or crepuscular behavior; Guthrie, Muntz & Pitcher (1993)), with visual adaptations being most apparent within fishes’ retinae (Land & Nilsson, 2012). Consequently, fishes may use photopic vision, which is mediated by cone photoreceptors, in well-lit conditions, or scotopic vision, which involves rod photoreceptors, under low-light conditions to mediate robust visual detection across their environmental light ranges.

The visual pigment sensitivity hypothesis proposes that the spectral sensitivity of a species tends to correspond to the spectrum of light available in its environment (Munz & McFarland, 1973). Previous studies have shown that the colors a species can visually distinguish are strongly related to the specific environmental niche it occupies (Bedore et al., 2013), resulting in spectral sensitivities that broadly align with available wavelengths of light (Denton & Warren, 1957; McFarland & Munz, 1975; Crescitelli, McFall-Ngai & Horwitz, 1985; Partridge, Archer & Lythgoe, 1988; Harrington, Hrabik & Mensinger, 2015). While low-light visual capabilities of freshwater fishes have often been examined using behavioral predator–prey studies (Richmond, Hrabik & Mensinger, 2004; Vinyard & O’Brien, 1976; Confer et al., 1978; Henderson & Northcote, 1985), physiological approaches can instead define the wavelengths to which fishes are most sensitive and the minimum light intensities required for visual detection. Such physiological constraints provide a foundation for interpreting visual habitat across spatial and temporal gradients, without directly inferring behavioral performance.

Walleye, *Sander vitreus*, are native to northern North American freshwater lakes and rivers (Scott & Crossman, 1973) and are the continent’s largest member of the *Percidae* family (Sloss, Billington & Burr, 2004). *S. vitreus* are a crepuscular fish (Scherer, 1976; Ryder, 1977) reputed for their low-light visual capabilities based on the presence of a well-developed *tapetum lucidum*, which acts to reflect unabsorbed photons back on to the photoreceptors, thus increasing visual sensitivity while lowering visual acuity (Moore, 1944; Ali & Anctil, 1968; Zyznar & Ali, 1975; Ali & Anctil, 1977). Among teleosts, visual acuity is strongly correlated with eye size (Caves, Sutton & Johnsen, 2017), and light capture is constrained by relatively fixed pupils (Cronin et al., 2014; Vinterstare et al., 2020). In *S. vitreus*, eye and lens diameter scale together, maintaining a constant eye-to-pupil ratio throughout development (Wahl, 1990). The presence of a tapetum lucidum increases visual sensitivity at the cost of acuity (Ollivier et al., 2004), making physiological measurements of visual sensitivity essential for understanding the optical foraging habitat of this species.

As *S. vitreus* mature, they undergo ontogenetic shifts in habitat and visual function (Eschmeyer, 1950; Braekevelt, Ward & McIntyre, 1989), with mature *S. vitreus* preferring light intensities around 28–30 lux (Scherer, 1976; Lester et al., 2004). While microspectrophotometry measurements place rod λmax at 533 nm (Ali & Anctil, 1977), this approach lacks the *in vivo* neural context captured by electroretinography (ERG), which measures the summed retinal response to light in intact eyes. Critically, while prior assessments have established rod sensitivity, they have failed to translate these thresholds onto depth-specific light fields across systems, thus leaving the detection thresholds that govern visual foraging unresolved.

Therefore, we sought to determine *S. vitreus* scotopic spectral sensitivity and to use ERG-derived thresholds together with light attenuation models to delineate depths and conditions permitting visual detection across aquatic habitats differing in optical clarity. Importantly, these data inform species-specific foraging models and link sensory capabilities to habitat use.

## Materials and Methods

### Fish collection and husbandry

*S. vitreus* were collected during the summer of 2015 *via* angling from Spirit Lake, WI on the St. Louis River estuary along the NE shore of Spirit Island at depths ranging from 3.0–3.5 m. Surface water temperatures on collection days ranged from 21.1–23.1 °C and Secchi depth was consistently ~0.7 m. Collected *S. vitreus* were temporarily stored and transported in a cooler (96.2 L) treated with 0.026% Stresscoat^®^ (Mars Fishcare North America Inc., Chalfont, PA, USA). During transport water was continually aerated *via* 8 cm Deluxe Bubble Disks (Penn Plax, Hauppauge, NY, US). *S. vitreus* were collected and transported in accordance with the State of Wisconsin Department of Natural Resources Scientific Collecting policy, permit No. SCP-NOR-073–0527. Portions of this text were previously published as part of a doctoral thesis (Keyler, 2018).

At the University of Minnesota Duluth, fish were housed in six 575 L mechanically, chemically, and biologically filtered (1500 Penn-Plax Cascade^™^ filters) recirculating tanks (Miller Manufacturing, Eagan, MN, US) that were kept in a 16.0 °C cold room (to minimize fungal and bacterial growth). Tanks were subjected to a 12 h: 12 h (L:D) photoperiod with a light intensity of ~3.48 × 10^13^ photons m^−2^ s^−1^ for the diurnal segment (0600 to 1,800 h), which emulates nautical twilight (Johnsen, 2012). Light intensity was measured using the International Light Technologies ILT1700 Research Radiometer (Peabody, MA, USA) and a SED033/F/HMR/W broadband silicon detector (Peabody, MA, USA). All fish husbandry and experimentation conformed to the University of Minnesota animal care protocols and were approved by the Institutional Animal Care and Use Committee Protocol ID: 1504–32496A in addition to the recommendations within the Guide for the Care and Use of Laboratory Animals of the National Institutes of Health.

### Additional animal care details

Fish were monitored daily for general health, feeding behavior, and water quality. Adult *S. vitreus* were fed Fathead minnows (*Pimephales promelas)* obtained from a local bait supplier three times per week. Because the fish were wild-caught and held short-term for terminal physiological measurements, no additional environmental enrichment beyond stable temperature, consistent photoperiod, and maintenance of optimal water quality was used. This approach is consistent with University of Minnesota Duluth IACUC protocol ID: 1504-32496A.

All experimental procedures were conducted under deep anesthesia (0.005% tricaine methanesulfonate) followed by immobilization with pancuronium bromide. No analgesia was administered because all procedures were performed under full surgical anesthesia and were terminal; analgesics are not appropriate for non-recovery neurophysiological recordings and may alter retinal responses. At the conclusion of each ERG trial, fish were euthanized by overdose of buffered tricaine (>250 mg L^−1^) followed by cervical transection, in accordance with the approved IACUC protocol.

### Electroretinograms

ERGs were conducted to determine the scotopic spectral sensitivity of *S. vitreus* (*n* = 9). Fish ranged from 31 to 44 cm and averaged 36.9 ± 1.4 cm (Mean ± S.E.) in total length (*L*_T_). Since fish were >30 cm *L*_T_ (Chevalier, 1973; tested range: 31 to 44 cm *L*_T_), aged 3–5 years (Olson, Wisconsin Department of Natural Resources, 2018, personal communication), they all should possess functional scotopic visual capabilities (Braekevelt, Ward & McIntyre, 1989; Vandenbyllaardt et al., 1991). Sex was not determined for experimental individuals. In walleye, sex-specific differences in habitat and depth use are primarily associated with the brief spawning period, whereas outside of spawning, males and females occupy largely overlapping depth distributions and experience similar ambient light environments (*e.g*., Matley et al., 2020). Because rod-mediated scotopic sensitivity reflects adaptation to long-term optical conditions rather than short-term reproductive behavior, sex was not expected to influence ERG-based measurements of scotopic spectral sensitivity. To limit any variation due to retinomotor movements, experiments were conducted between 1,200 and 1,800 h to account for endogenous timekeeping mechanisms, or internal biological clocks (Cahill & Besharse, 1995; Li & Dowling, 1998).

Fish were transferred from their holding tanks to the experimental room. All experimental procedures were conducted in a dark room illuminated by dim red light (15 W bulbs fitted with Kodak GBX-2 dark red safelight filters), allowing researchers to navigate without interfering with scotopic adaptation. Before testing, fish were anesthetized by immersion in a 0.005% tricaine solution within a 50 L recirculating holding tank filled with a chilled (16 °C; 420 W Teco SeaChill Aquarium Chiller, Teco model SCTR20, Ravenna, Italy) buffered d_i_H_2_0 solution (1.1% potassium phosphate monobasic and 2.5% sodium phosphate dibasic; Sigma Chemical Co., St. Louis, MO, USA). To ensure that the fish were properly anesthetized, a tail pinch was administered prior to an intramuscular injection of 0.01% pancuronium bromide (600 μg kg^−1^).

Anesthetized and immobilized fish were secured between moistened sponges in an acrylic experimental tank (37 × 15 × 11 cm) within a black opaque Faraday cage (77 × 67 × 96 cm). *S. vitreus* were submerged to the ventral border of the eye and received a chilled (16 °C), buffered, tricaine (0.005%) water solution that was pumped over the gills *via* an intraoral tube to maintain anesthesia. A small incision (<1 mm) was made at the limbus of the eye with a microsurgical knife (Sharpoint^™^, Reading, PA, USA) and an Ag-AgCl 0.13 mm diameter (Cat. No. 781500; A–M Systems, Inc., Carlsborg, WA, USA) recording electrode was inserted within the vitreous body of the eye behind the lens. A reference electrode with the same specifications was positioned subdermally between the nares of the fish. The ERG response was amplified (1000x; model DAM50; World Precision Instrument, Inc., Sarasota, FL, USA) and filtered with a 10 Hz low pass filter and 10 kHz high pass filter. Under this electrode configuration and 1,000× amplification, b-wave amplitudes were in the millivolt range, consistent with previous reports in large freshwater teleosts, including walleye, using comparable vitreous electrode configurations (Harrington, Hrabik & Mensinger, 2015). All data was recorded using a PowerLab 4SP data acquisition system (AD Instruments, Castle Hill, NSW, Australia) and analyzed using LabChart7 software (v.7.3.7, ADInstruments, Castle Hill, NSW, Australia).

**Figure 1:**
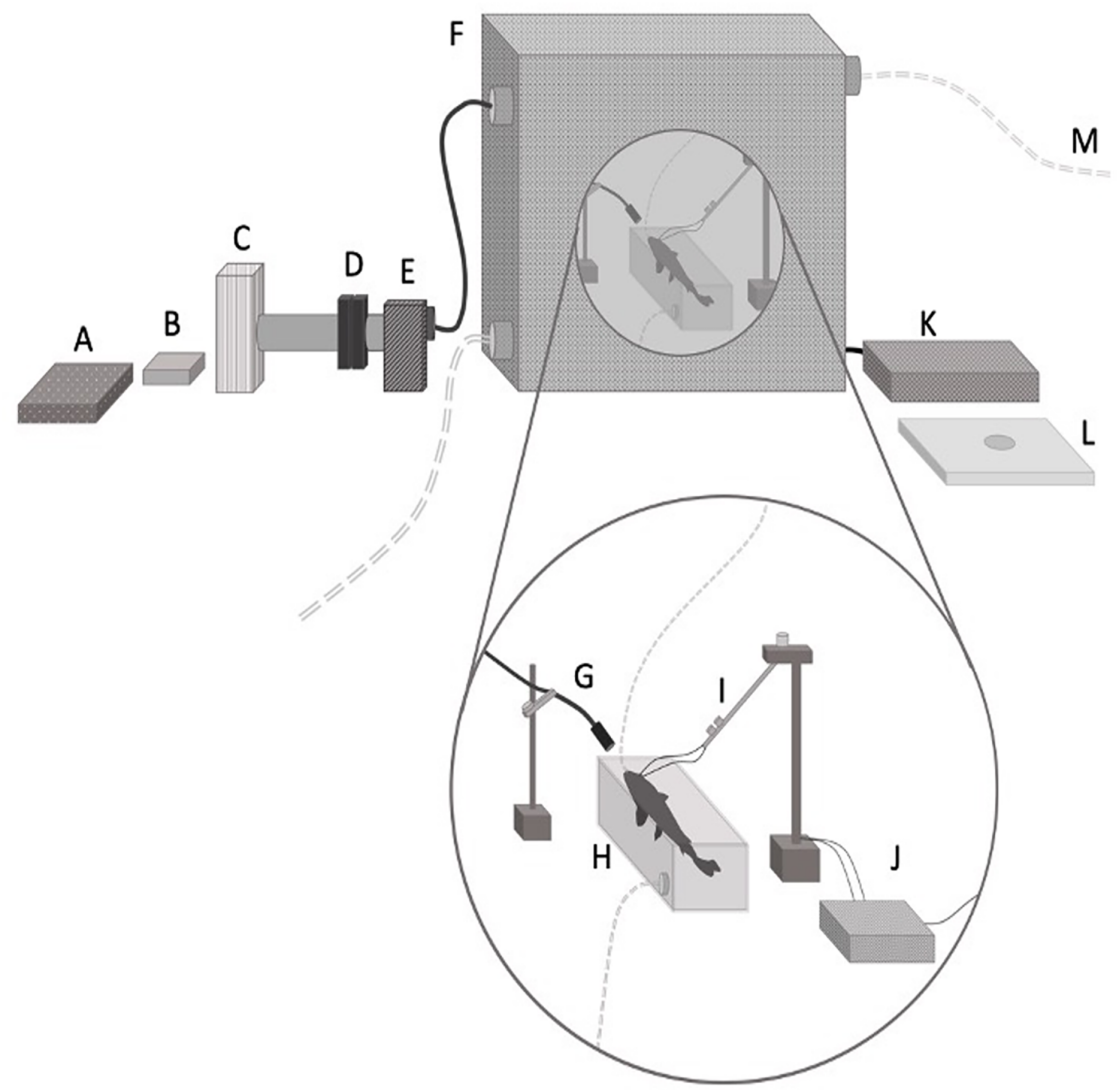
Schematic of electroretinography setup. From left to right: (A) Constant current power supply, (B) electric shutter control, (C) 100 W quartz-tungsten halogen lamp, (D) neutral density filters, (E) monochromator, (F) Faraday cage (77 × 67 × 96 cm), (G) fiber optic light pipe, (H) acrylic experimental tank (37 × 15 × 11 cm), (I) recording and reference electrodes, (J) signal amplifier, (K) PowerLab, (L) personal computer, (M) chilled (16 °C) buffered water lines.

After electrode insertion, *S. vitreus* were dark-adapted until no alpha-wave component of the light-evoked response was visible (30–60 min). A 100 W quartz-tungsten halogen lamp (Newport model 6333, Stratford, CT, USA) powered by a constant current power supply (Newport model 68938, Stratford, CT, USA) provided the 200 ms light stimulus for the ERG (Fig. 1). Square-wave light pulses (3.0, 3.0 and 5.0 ms delay, rise and fall times, respectively) were regulated by a controller (Oriel^®^ Instruments model 76995, Stratford, CT, USA), which controlled an electric shutter (Oriel^®^ Instruments model 76994) responsible for modulating stimulus duration. The light from the lamp transited through a monochromator (Newport model 77250), passed through a series of 0.1 to 5.0 neutral density filters (Newport FSR-OD series filters: wavelength range 400 to 900 nm, neutral transmission 400 to 700 nm) and into a fiber optic light pipe (Newport model 77632) positioned 3 cm away to illuminate the entire fish eye.

**Figure 2:**
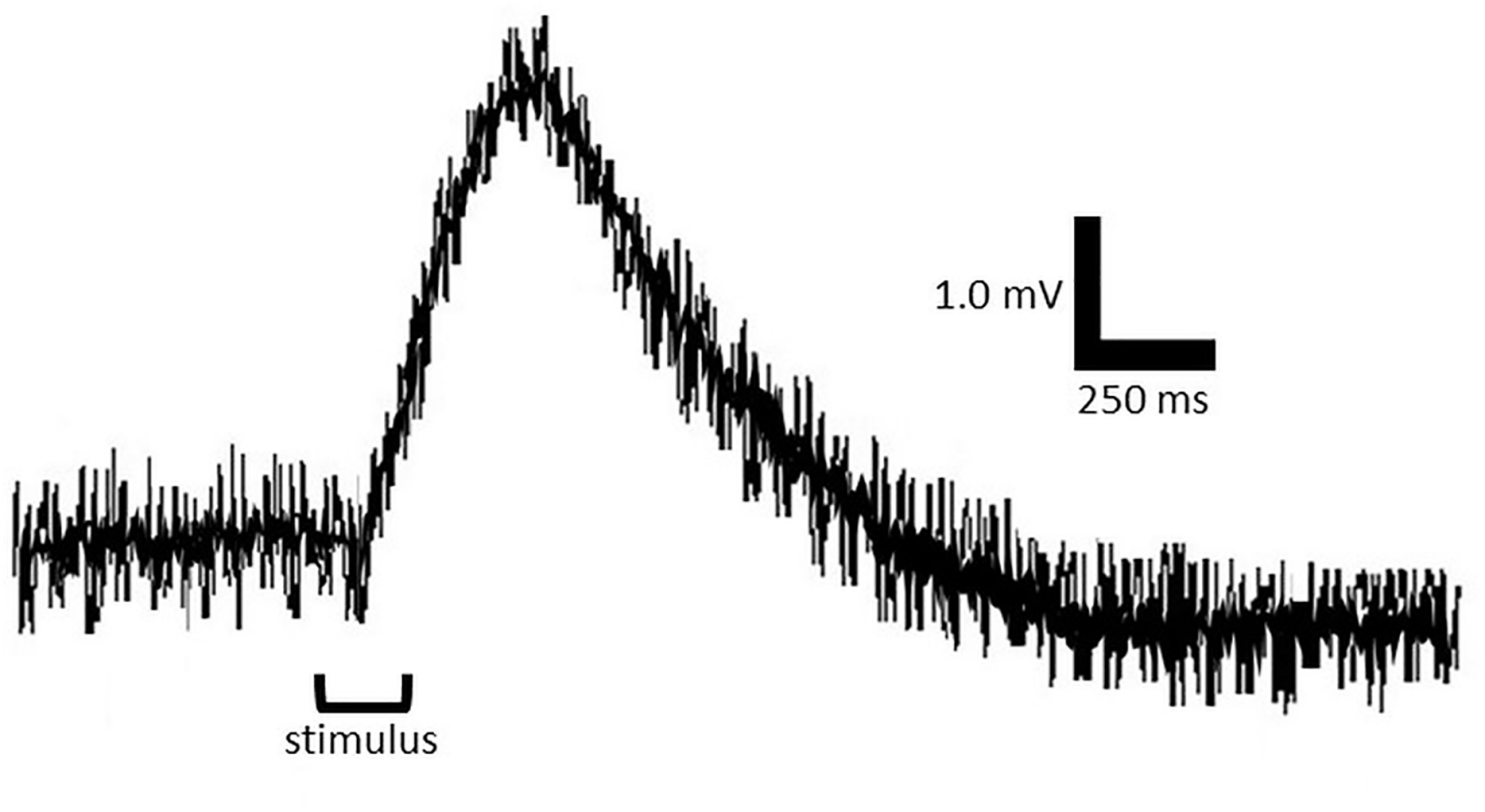
Representative electroretinogram (ERG) response from an adult *Sander vitreus* to a 200 ms flash of 550 nm light. The waveform shows a clear b-wave under dark-adapted conditions. The absence of an a-wave supports that the response is rod-mediated and reflects scotopic visual sensitivity.

The stimulus consisted of a 200 ms flash of monochromatic light of wavelengths starting at 400 nm and proceeding in 25 nm increments to 700 nm. The presentation order of the wavelengths was randomly determined before each trial. An interstimulus duration of 30 s was sufficient to achieve the same b-wave amplitude for consecutive flashes and avoid photobleaching. The response of the dark-adapted retina was determined by measuring the amplitude of the b-wave from baseline to peak. Shorter wavelength light (≤425 nm) consistently elicited lower b-wave amplitudes; therefore, the criterion response was initially determined by measuring the b-wave amplitude in response to a 400 nm stimulus. Under these scotopic, dark-adapted conditions, the recorded b-wave is widely accepted to reflect rod-driven retinal activity transmitted *via* bipolar and Müller cells. The absence of an a-wave, which is generated by cone photoreceptors under photopic conditions, confirms that the ERG responses represent scotopic vision (Fig. 2). The spectral sensitivity curve was generated *via* neutral density filters that were used to reduce light intensity for tested wavelengths until the ±10% of the irradiance necessary to invoke the criterion response at each tested wavelength was obtained. It is important to note that retinal sensitivity values are not representative of absolute threshold values at each tested wavelength. Values should be considered relative thresholds as retinal sensitivity may be greater than what we can detect *via* ERG technique.

### Visual depth profile calculation

The minimum light intensities determined from the scotopic spectral sensitivity calculations and solar/lunar surface irradiances were used to calculate the maximum depths sufficient to elicit the criterion b-wave response (Eq. (1)), defined as the irradiance threshold required to trigger bipolar cell depolarization under rod-dominated, dark-adapted conditions. These estimates represent the limits of scotopic visual function and do not assess potential cone contributions at shallower depths, where mixed rod-cone input may occur. Calculations follow the methods of Keyler et al. (2015), which apply the Beer-Lambert equation (Hutchinson, 1957):

(1) $${I_x} = {I_o}{e^{ - kx}}$$

where surface irradiance, ${I_{o\; }} = 9.57\; \times \; {10^{19}}$ photons m^−2^ s^−1^ is the surface intensity from solar radiation in summer at noon for Lake Superior (Fahnenstiel, Schelske & Moll, 1984). Surface intensity values from solar radiation for Lake Superior were used since all *S. vitreus* were captured from the mouth of the St. Louis river estuary, which is the confluence of the St. Louis River with Lake Superior. Unfortunately, Lake Superior surface irradiance from lunar radiation is reported in lux, which correlates to human vision and is not appropriate to use for fish vision calculations. Therefore, following Harrington, Hrabik & Mensinger (2015), the surface irradiance from lunar radiation, ${I_{o\; }} = 1.35\; \times \; {10^{10}}$ photons m^−2^ s^−1^ was used from Cramer et al. (2013) who reported wavelength specific surface irradiance for moonlight (waning gibbous, 2 days post-full) in Arizona. Spectral irradiance attenuation coefficients reported by Jerome, Bukata & Bruton (1983) were used to determine various vertical attenuation coefficients *k* for calculating light at depth. Light attenuation *k* varies within systems due to light-absorbing particulates such as dissolved organic carbon compounds and suspended sediments (Guthrie, Muntz & Pitcher, 1993). The vertical attenuation coefficients of k_PAR_ = 0.3 and 0.5 were derived from Lake Superior data representing a clear-water system, while attenuation coefficients from Lake Ontario of k_PAR_ = 0.8 and 1.2 were used to represent a higher turbidity system (Jerome, Bukata & Bruton, 1983).

### Statistical analysis

Statistical tests were performed using JMP software (v.10.0, Statistical Analysis System Institute Inc., Cary, NC, USA). All data was tested for and passed the Shapiro-Wilks normality test and Brown-Forsythe equal variances test. An ANOVA was performed to determine the effect of stimulus wavelength on irradiance needed to evoke a criterion response. A Tukey’s HSD *post-hoc* test was performed to examine pairwise differences in irradiance at each stimulus wavelength. All statistical tests used a significance value of α = 0.05.

## Results

### Visual spectral sensitivity

**Figure 3:**
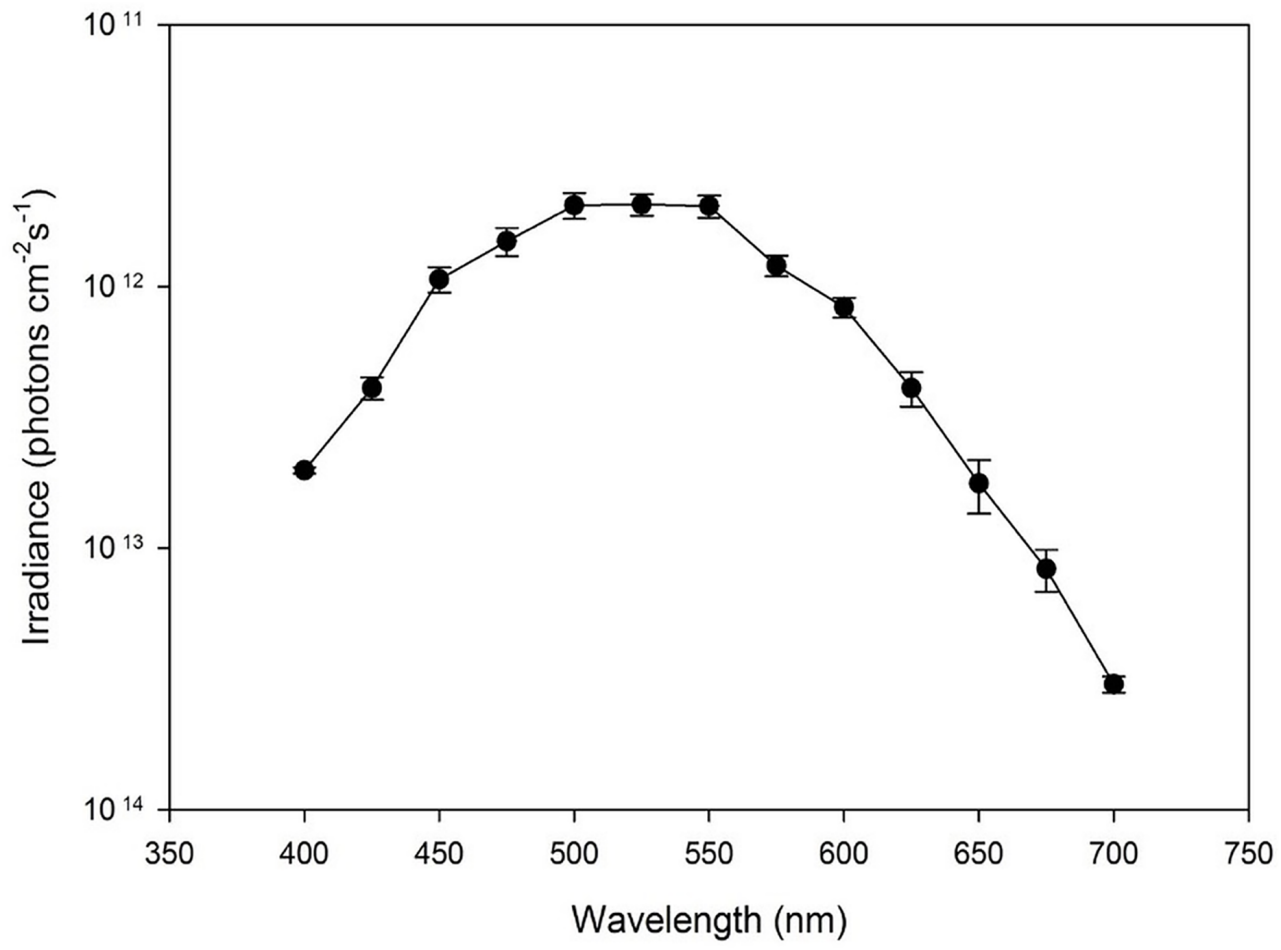
The average irradiance (photons cm^−2^ s^−1^) needed to invoke the criterion response *vs*. wavelength (nm) for *S. vitreus* (*n* = 9). Error bars represent ± S.E.

The irradiance (photons cm^−2^ s^−1^) necessary to evoke a criterion response at each wavelength was used to create the scotopic spectral sensitivity curve for the dark-adapted *S. vitreus* retina. The spectral sensitivity followed a parabolic curve from 400 to 700 nm with minimum sensitivity occurring at 700 nm. There was a significant effect of wavelength on the average irradiance (photons cm^−2^ s^−1^) needed to invoke the criterion response (Fig. 3; ANOVA, F₁₂,₁₀₄ = 7.90, *p* < 0.0001). *S. vitreus* displayed peak sensitivity from 500 to 550 nm, defined by a significant increase in sensitivity from 475 to 500 nm (Tukey’s HSD, *p* < 0.05), no significant changes between 500 to 550 nm, and a significant decrease from 550 to 575 nm (Tukey’s HSD, *p* < 0.05).

### Visual depth profiles

**Figure 4:**
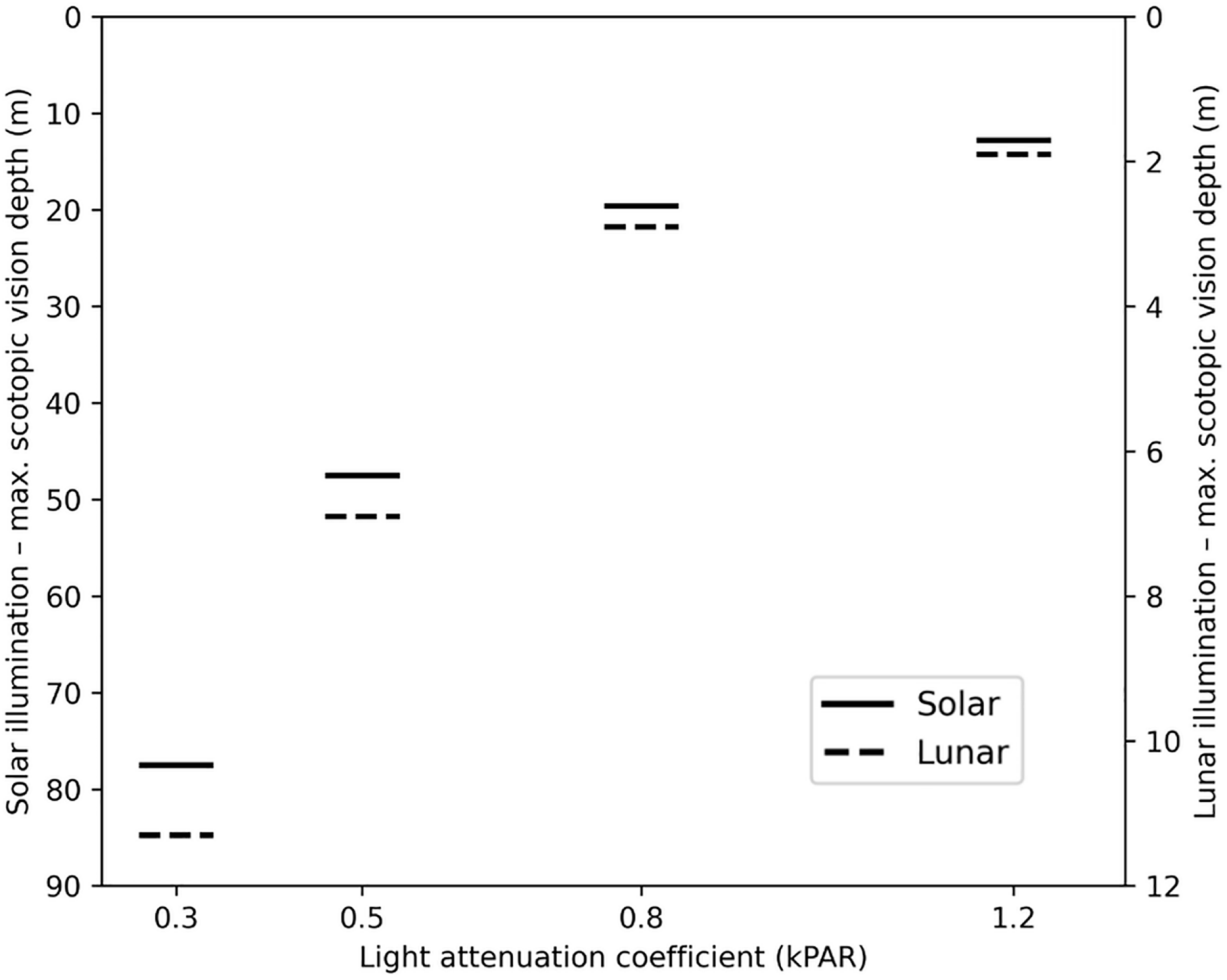
Maximum depth (m) for scotopic vision in *Sander vitreus* across a range of light attenuation values (kPAR) under solar and lunar illumination. Horizontal bars indicate the maximum depth at which sufficient irradiance is available to elicit the criterion b-wave response based on peak rod-based spectral sensitivity, for four water clarity conditions (kPAR = 0.3, 0.5, 0.8, 1.2). Solid lines (left y-axis) represent modeled detection depths under solar illumination (summer midday), whereas dashed lines (right y-axis) represent detection depths under lunar illumination (waning gibbous moon, 2 days post-full).

To determine the maximum depth where sufficient irradiance exists to elicit the criterion ERG response, “visual depth profiles” were created to approximate the depth within a given body of water where light detection may be possible under scotopic conditions. These depths reflect the limits of light detection under rod-driven, low-light conditions and do not account for potential cone contributions at shallower depths. Results indicate dark-adapted *S. vitreus* under daytime conditions (noon) can detect light as deep as 77.5 m (kPAR = 0.3) and 47.5 m (kPAR = 0.5) respectively (Fig. 4). With increased turbidity of kPAR = 0.8 and 1.2, the maximum depth of scotopic visual detection decreased to 19.6 and 12.8 m, respectively (Fig. 4). At night, *S. vitreus* can detect light to depths of 11.3, 6.9, 2.9, and 1.9 m for respective kPAR values of 0.3, 0.5, 0.8 and 1.2 (Fig. 4).

## Discussion

ERG revealed that the peak scotopic spectral sensitivity of *Sander vitreus* (walleye) occurs between 500 and 550 nm. Based on the scotopic spectral sensitivity results, we estimated the maximum depths at which rod-mediated visual detection may occur under solar and lunar illumination in different aquatic systems. These depth estimates reflect the maximum depths at which light is sufficient to elicit a rod-mediated ERG response, indicating the potential for scotopic vision. Under midday solar illumination, dark-adapted *S. vitreus* could detect light to at least 77.5 m in a system where kPAR = 0.3 and 12.8 m in a turbid system where kPAR = 1.2. Under lunar illumination (waning gibbous, 2 days post-full), detection depths decreased to 11.3 and 1.9 m for those same respective kPAR values. Numerous studies have noted that *S. vitreus* are adapted for low-light foraging, in part due to the presence of a tapetum lucidum, a reflective retinal structure that enhances scotopic sensitivity by increasing photon capture (Ryder, 1977; Kelso, 1978; Wahl, 1994; Barton, 2011). Low-light vision depends on the scotopic spectral sensitivity of the retina, the transmission properties of the ocular media, and the spectral characteristics of the retinal visual pigments. The presence of a tapetum lucidum is known to increase visual sensitivity at the expense of visual acuity (Ollivier et al., 2004), underscoring the importance of physiological measures when evaluating limits of visual detection. In tapetal species such as *S. vitreus*, this retinal specialization enhances sensitivity under low-light conditions while reducing spatial resolution. These established tradeoffs provide important context for interpreting scotopic visual sensitivity, without implying direct links between retinal physiology and behavioral habitat selection.

Ali, Ryder & Anctil (1977) previously investigated the scotopic visual pigment in *S. vitreus* (formerly *Stizostedion vitreum vitreum*) *via* microspectrophotometry and found that rods absorb maximally at ~533 nm. These findings align with our ERG scotopic spectral sensitivity data, which determined peak sensitivity from 500 to 550 nm. Microspectrophotometry measures photopigment absorbance in isolated photoreceptors, whereas ERG reflects the summed retinal response to light in the intact eye; agreement between these results supports our interpretation that the ERG signal primarily reflects rod-based vision. Additionally, Ali, Ryder & Anctil (1977) reported cone absorption peaks at ~560 nm for single cones and ~630 nm for double cones, supporting the interpretation that the ERG responses were dominated by rod-mediated activity under dark-adapted conditions, while acknowledging that some cone contribution cannot be fully excluded due to spectral overlap among photoreceptor classes.

Our findings are consistent with those previously reported by Ali, Ryder & Anctil (1977). However, unlike microspectrophotometry, ERG measures the *in vivo* neural response of the retina, providing insight into the cellular depolarization that occurs during light detection. Michels et al. (2025) also used ERG to examine scotopic spectral sensitivity in juvenile *S. vitreus* and reported a nearly identical peak range (500–550 nm) to that found in our adult fish. Our results therefore, provide independent validation of these findings using a different population and experimental conditions, strengthening confidence in this sensitivity estimate for *S. vitreus*. Although peak spectral sensitivity was similar across life stages, the irradiance thresholds required to elicit a criterion response in adult *S. vitreus* were approximately one order of magnitude lower than those reported for juveniles by Michels et al. (2025), indicating higher absolute scotopic sensitivity in adults.

Spectral sensitivity can assist in making inferences about a fish’s visual depth capabilities. Mature *S. vitreus* prefer depths associated with light intensity ranging from 8 to 68 lux (Lester et al., 2004) and temperatures between 20 °C and 24 °C (Hokanson, 1977). Because our dataset includes only scotopic ERG thresholds, visual function at shallower depths—where cone input may be possible—is beyond the scope of this analysis. Under scotopic (rod-dominated) conditions, *S. vitreus* can detect solar light to depths of at least 77.5 m (kPAR = 0.3), 47.5 m (kPAR = 0.5), 19.6 m (kPAR = 0.8), and 12.8 m (kPAR = 1.2) under optimal daytime conditions (high noon, minimal cloud cover). Under lunar conditions (waning gibbous, 2 days post-full), scotopic spectral sensitivity results indicate that *S. vitreus* can detect moonlight to depths of 11.3, 6.9, 2.9, and 1.9 m at the same respective kPAR values. While these estimates represent thresholds for retinal activation under scotopic conditions, functional capabilities such as contrast detection, image formation, or prey capture likely require higher irradiance and may involve cone input in shallower waters. Michels et al. (2025) used comparable scotopic ERG data to model prey detection distances in juvenile *S. vitreus*. Although our adult data suggest higher sensitivity, we did not attempt to model detection distances. Instead, we focus on defining the physiological limits of scotopic light detection, which may help guide future work on depth use and foraging ecology.

Previous studies that have investigated the spatial and temporal aspects of *S. vitreus* distribution have reported that the observed depths occupied are shallower than the depth estimates at which vision is possible (Table 1). For example, within mesotrophic bodies of water (4 m Secchi depth) where *S. vitreus* are most abundant (Regier, Applegate & Ryder, 1969; Kitchell et al., 1977; Leach et al., 1977; Schupp, 1978; Lester et al., 2004) the *S. vitreus* depth range is from 12–18 m for a summer’s day at noon. This study suggests *S. vitreus* could use vision to depths of at least 47.5 m within a comparable mesotrophic system (k_PAR_ = 0.5), almost 30 m deeper than the predictions from Lester et al. (2004). However, it is important to reiterate that these results represent the approximate maximum depths where light detection is possible as additional illumination may be needed to mediate predator-prey interactions. Michels et al. (2025) found that most prey orientation and capture attempts in age-0 *S. vitreus* occurred at ~0.05–1 lx, corresponding to light levels present between nautical and civil twilight. While their trials involved juveniles under controlled conditions, the modeled lunar illumination depths for adults overlap with the light environments in which juveniles have been shown to forage. Although irradiance thresholds required to elicit a criterion response differ between adults and juveniles, these modeled depths remain above juvenile detection thresholds, resulting in overlap in ecologically relevant light environments. Differences in eye size, retinal structure, and habitat use between juveniles and adults mean that the exact relationship between modeled depth limits and realized adult foraging performance remains to be tested in the field.

**Table 1:** Predicted maximum depths derived from *S. vitreus* visual depth profiles. Summary of studies comparing water type, maximum water depth (m), and observed fish depths (m) during day and night with modeled maximum depths (m) for scotopic retinal light detection under summer conditions. Predicted maximum depths are derived from *S. vitreus* visual depth profiles and represent physiological thresholds for retinal activation, not functional vision or realized foraging behavior.

Species	Author	Water type	Max water depth	Day-actual fish depth	Day-predicted max. depth	Night-actual fish depth	Night-predicted max. depth
*S. vitreus*	Lester et al. (2004)	Mesotrophic	20	12–18^‡^	47.5	–	–
	Byrne, Stich & Foster (2009)	Mesotrophic	51	10^†^	47.5	7^†^	6.9
	Williams (1997)	Mesotrophic	76	6	47.5	–	–
	Kelso (1978)	Oligotrophic	30	5–10	77.5	–	–
	Haxton et al. (2015)	Oligotrophic	200	6–12	77.5	–	–
	Dendy (1948)	Eutrophic	53	3^‡^	12.8	–	–
	Clark-Kolaks (2009)	Eutrophic	18	6^†^	12.8	–	–
	Holt et al. (1977)	Eutrophic	25	5	12.8	–	–
*P. flavescens*	Rudstam & Magnuson (1985)	Mesotrophic	36	7	–	–	–
	Rudstam & Magnuson (1985)	Oligotrophic	20	18	–	12	–
	Lyons & Magnuson (1987)	Oligotrophic	20	3–4	–	–	–

**Notes**:

† max. depth.

‡ predicted depth.

Dashes (–) indicate unreported depth values.

Additionally, abiotic factors (*e.g.*, oxygen, temperature) and biotic factors (*e.g.*, prey/predator density; McFarland (1986), De Robertis (2002), Boscarino et al. (2010)) will influence the actual depths occupied within different systems. Fishes feed where prey are located and will forage in suboptimal light conditions if necessary to optimize prey capture (Crowder & Cooper, 1982). In order for *S. vitreus* to use visual cues during predator-prey interactions, they may need to forage at shallower depths where both increased illumination and prey fishes, such as *P. flavescens*, are found (Table 1). While there is limited information on nocturnal *S. vitreus* distribution, the maximum depths at which light detection is possible more closely align with maximum fish depth. As nocturnal foraging relies predominantly on rod photoreceptors, the depth distribution model suggests that nocturnal depths may be more accurate. However, additional information on *S. vitreus* nocturnal depth distribution will be needed to validate the model.

The scotopic visual sensitivity determined here for *S. vitreus* indicates physiological adaptation to low-light environments and defines the limits under which visual detection may remain possible under rod-dominated conditions. Visual physiology studies using electroretinography can identify the wavelengths and minimum light intensities required to elicit retinal responses, and when combined with light attenuation models, provide a framework for interpreting potential visual habitat across spatial and temporal gradients. These results emphasize that ERG-derived thresholds describe the physiological capacity for light detection rather than realized foraging performance, which will also be influenced by ecological context and behavior. Integrating physiological constraints with field-based observations remains an important avenue for refining predictions of habitat use in low-light freshwater systems.

## Conclusions

Electroretinography revealed that scotopic spectral sensitivity in adult *Sander vitreus* peaks between 500 and 550 nm, consistent with previous microspectrophotometric and ERG studies of this species. When combined with light attenuation models, these measurements indicate the depths at which scotopic (rod-dominated) retinal responses may occur under solar and lunar illumination across a range of water clarities. These depth estimates represent upper physiological limits for light detection rather than thresholds for functional vision or foraging performance. Accordingly, ERG-derived scotopic thresholds describe the capacity for retinal activation under low-light conditions and should be interpreted independently from realized habitat use or prey capture behavior.

## Supplemental Information

10.7717/peerj.21156/supp-1 Supplemental Information 1 Summary of studies comparing water type, maximum water depth (m), and actual fish depths (m) for day/night to our predicted maximum depths (m) where visually mediated behavior may be possible during summer. Predicted max. depths values are from *S. vitreus* visual depth profiles. Dashes (-) indicate unreported depth values.

10.7717/peerj.21156/supp-2 Supplemental Information 2 ARRIVE checklist.

## Additional Information and Declarations

### Data Availability

The following information was supplied regarding data availability:

The raw data is available in the Supplemental File.
